# Study on the improvement of cognitive deficits in APP/PS1 mice by danggui shaoyao san and its disassembled prescriptions through modulation of the gut microbiota

**DOI:** 10.3389/fmicb.2025.1620784

**Published:** 2025-07-31

**Authors:** Xingduo Liu, Chaoqun Sun, Yuqiong Dai, Feifei Duan, Tianzhen He, Menglu Zhen, Enxi Liang, Shuting Zhang, Yun Xia, Nianchun Hu, Ruoting Zhan, Dong Deng, Sijun Liu

**Affiliations:** ^1^School of Pharmaceutical Sciences, Guangzhou University of Chinese Medicine, Guangzhou, China; ^2^Shenzhen Luohu Traditional Chinese Medicine Hospital, Shenzhen, China; ^3^Department of Neurology, 921 Hospital of Joint Logistics Support Force, Changsha, China; ^4^Science and Technology Innovation Center, Guangzhou University of Chinese Medicine, Guangzhou, China; ^5^Key Laboratory of Chinese Medicinal Resource from Lingnan, Guangzhou University of Chinese Medicine, Guangzhou, China

**Keywords:** Alzheimer’s disease, APP/PS1 mice, Danggui Shaoyao San, gut microbiota, microbiota-gut-brain axis

## Abstract

**Background:**

Alzheimer’s disease (AD), a neurodegenerative disorders linked to gut microbiota dysbiosis, may benefit from Traditional Chinese Medicine (TCM) interventions.

**Objectives:**

Danggui Shaoyao San (DSS), a classic traditional Chinese Medicine (TCM) formula. This study investigated whether Danggui Shaoyao San and its disassembled prescriptions could improve cognitive deficits in APP/PS1 mice by modulating the structure of the gut microbiota, thereby providing a theoretical basis for AD treatment and the further development and application of Danggui Shaoyao San.

**Methods:**

Forty APP/PS1 and eight C57BL/6 mice were divided into six groups: DSS (6.4 g/kg/d), QDW (4.6 g/kg/d), DW (1.8 g/kg/d), GV971 (positive control, 40 mg/kg/d), model (saline), and control (saline). After 60 days of treatment, the mice underwent behavioral testing in the open field, novel object recognition, and water maze. Gut microbiota composition, diversity, and function were then analyzed by 16S rRNA sequencing.

**Results:**

The results of Behavioral experiment indicate that Danggui Shaoyao San and its disassembled prescriptions can ameliorate spatial memory deficits (Morris water maze), enhance recognition memory (novel object recognition), and reduce anxiety-like behaviors (open field test), with the DSS group demonstrating the most pronounced effects. In addition, through 16S sequencing analysis we predicted DSS and its disassembled prescriptions reduced harmful bacteria (Firmicutes, Akkermansia) while increasing beneficial bacteria (Bacteroidetes, Bifidobacterium, Lactobacillus). DSS restored microbial diversity closest to healthy controls, evidenced by elevated Chao1/Shannon indices and reduced Simpson index. Beta diversity revealed structural divergence between treatment and model groups. Functional predictions highlighted enriched pathways (D-glutamine metabolism, bile acid biosynthesis) and suppressed antibiotic biosynthesis.

**Conclusion:**

Danggui Shaoyao San and its disassembled prescriptions ameliorate AD-related cognitive impairment and gut dysbiosis, enhance microbial diversity, and modulate metabolic pathways, supporting their therapeutic potential via gut-brain axis regulation. This study elucidates the multi-target mechanisms of DSS in AD treatment, advancing TCM rationalization for neurodegenerative disorders.

## 1 Introduction

Alzheimer’s disease (AD), a progressive neurodegenerative disorder, remains a leading cause of dementia with limited therapeutic options. It predominantly affects individuals over the age of 65 and is characterized by progressive cognitive decline, including deteriorating memory and impaired logical reasoning (Chen and Zhang, 2022; Yang et al., 2024).

Growing evidence highlights the role of gut microbiota dysbiosis in AD pathogenesis, mediated through the microbiota-gut-brain axis (MGBA), which regulates neuroinflammation, amyloid-beta (Aβ) deposition, and cognitive decline (Wang et al., 2016; Kim et al., 2021; Grabrucker et al., 2023). MGBA drives the onset and progression of AD through core pathological processes such as gut barrier function damage induced by gut microbiota dysbiosis, peripheral inflammatory mediators (such as LPS and SCFAs) entering the bloodstream, increased blood-brain barrier permeability, activation of neuroinflammation, and Aβ deposition and Tau protein phosphorylation (Harach et al., 2017; Gao et al., 2019; Liu et al., 2019; Chandra et al., 2023).

Traditional Chinese Medicine (TCM) formulas, such as Danggui Shaoyao San (DSS), have demonstrated neuroprotective effects in AD models by reducing Aβ aggregation and modulating neuroinflammatory pathways (Song et al., 2021; Wang et al., 2023; Cheng et al., 2024; Wu et al., 2024). However, the mechanisms underlying DSS’s therapeutic efficacy, particularly its interaction with gut microbiota, remain poorly understood.

Recent studies suggest that TCM formulas exert systemic effects by restoring gut microbial homeostasis, yet the contribution of specific herbal combinations within multi-component formulas remains unexplored. DSS, which comes from the Synopsis of the Golden Chamber, composed of six herbs (Angelica sinensis (Oliv.) Diels, Paeonia lactiflora Pall., Atractylodes macrocephala Koidz., Ligusticum chuanxiong Hort., Poria cocos (Schw.) Wolf., and Alisma orientale (Sam.) Juz.), it can be divided into two functional clusters according to its medicinal properties: Poria cocos (Schw.) Wolf. and Alisma orientale (Sam.) Juz. in the DW group are sweet and light in nature, while Ligusticum chuanxiong Hort., Angelica sinensis (Oliv.) Diels, Atractylodes macrocephala Koidz. and Paeonia lactiflora Pall. in the QDW group are not sweet and light in nature. The whole formula has shown AD-modifying properties, whether its efficacy arises from synergistic interactions between these clusters or individual components is unknown. This knowledge gap impedes the rational optimization of TCM prescriptions for precision medicine.

In APP/PS1 mice, the expression of amyloid precursor protein and human presenilin 1 is driven by the prion protein promoter. This enables the mice to mimic several clinical and pathological features of AD, including Aβ deposition, inflammatory cytokine responses, Tau protein hyperphosphorylation, neurofibrillary tangles, and neuronal loss, with these pathological changes worsening with age and ultimately leading to cognitive impairment (Jankowsky et al., 2004; Long et al., 2021; Mifflin et al., 2021). Therefore, the APP/PS1 mouse is a well-established model of AD that is widely used.

Therefore, this study aimed to investigate whether DSS and its disassembled prescriptions (QDW and DW) improve cognitive deficits in APP/PS1 mice and elucidate the role of gut microbiota modulation in this process. We hypothesized that DSS and its components exert therapeutic effects, at least partially, by restoring gut microbial homeostasis and function. Utilizing behavioral assessments (Open Field Test, Novel Object Recognition, Morris Water Maze) combined with 16S rRNA gene sequencing and functional prediction of the gut microbiota, we assessed cognitive function, microbial composition, diversity, and associated metabolic pathways. Our findings provide mechanistic insights into the therapeutic potential of DSS via the gut-brain axis and contribute to the rationalization of TCM for neurodegenerative disorders.

## 2 Materials and methods

### 2.1 Animal sorting

APP/PS1 mice were selected as a well-established model of AD pathology, while C57BL/6 served as a healthy control. 40 male APP/PS1 mice of SPF grade (4 months old) and eight male C57BL/6 mice of SPF grade (4 months old) were purchased from Hangzhou Ziyuan Experimental Animal Science and Technology Co, Ltd, Animal License No. SCXK (zhe) 2024-0004. All the experimental animals were housed in the Animal Experiment Center of Guangzhou University of Chinese Medicine (Mice were maintained at 24 ± 2°C with 55 ± 1% relative humidity and a 12/12 h light–dark cycle, with free access to standard feed and water, and the bedding was changed regularly to keep the environment of mice rearing clean. After entering the barrier environment, all mice underwent a 7 days acclimation period to adjust to the new housing conditions. After this period, 40 APP/PS1 mice were divided into five groups—DSS, QDW, DW, GV971, and MODEL according to the completely randomized grouping method, with 8 mice in each group. The control group (CON) consisted of eight C57BL/6 mice. The animal experiment protocol was approved by the Animal Care and Use Committee of Guangzhou University of Chinese Medicine in August 2024 (Approval No. 20240820004). All animal husbandry and experiments were conducted in strict accordance with the Institutional Guidelines for the Care and Use of Laboratory Animals of the Animal Experimentation Center of Guangzhou University of Chinese Medicine.

### 2.2 Preparation of DSS, QDW, DW, and GV971

The herbs used in the Danggui Shaoyao San used in this experiment were purchased from Guangdong Daxiang Traditional Chinese Medicine Pharmaceutical Co. Ltd, including Angelica sinensis (Oliv.) Diels (product lot number: HX24M02), Paeonia lactiflora Pall. (product lot number: HX24F01), Atractylodes macrocephala Koidz. (product lot number: HX24F01), Ligusticum chuanxiong Hort. (product lot number: HX24G02), Poria cocos (Schw.) Wolf. (product lot number: HX24G05), and Alisma orientale (Sam.) Juz. (product lot number: HX23B01). It is based on the drug ratios in the classic Chinese medicine prescription “The Synopsis of the Golden Chamber” and has been optimized according to previous studies that have shown efficacy in neurodegenerative models (Cheng et al., 2024). The DSS group was made according to the ratio: Angelica sinensis (Oliv.) Diels, Paeonia lactiflora Pall., Atractylodes macrocephala Koidz., Ligusticum chuanxiong Hort., Poria cocos (Schw.) Wolf. and Alisma orientale (Sam.) Juz. = 3:16:4:8:4:8. The ratio of Angelica sinensis (Oliv.) Diels: Paeonia lactiflora Pall., Atractylodes macrocephala Koidz., Ligusticum chuanxiong Hort. = 3:16:4:8 in the QDW group, and Poria cocos (Schw.) Wolf.: Alisma orientale (Sam.) Juz. = 4:8 in the DW group. Weighed Chinese herbal medicinal herbs were put into a round-bottomed flask, and eight times the amount of distilled water was added to the flask, soaked for 30 min, and then the filtrate was boiled for 1 h and collected, and the filtrate was then poured into a different container, and six times the amount of distilled water was poured into the dregs of the medicinal herb, and boiled. Pour the filtrate into another container, pour six times the volume of distilled water into the dregs, boil for 1 h, collect the filtrate of the two decoctions, concentrate, and store in the refrigerator at −20°C in separate packages.

Dissolve the positive drug Sodium Oligomannate Capsules (Shanghai Lvgu Pharmaceutical Co., Ltd.) in distilled water to make a suspension, and store in the refrigerator at −20°C in separate units, which is named GV971 group.

### 2.3 Drug treatment

All mice were administered at the end of the acclimatization period, and based on the results of the preliminary literature research (Yang et al., 2021), Danggui Shaoyao San was administered to the DSS group (6.4 g/kg/d), the QDW group (4.6 g/kg/d), and the DW group (1.8 g/kg/d). Positive drug group was given Sodium Oligomannate Capsules at a dose of 40 mg/kg/d (Bosch et al., 2024); the MODEL group and the CON group were given an equal amount of saline at 0.1 mg/10 g. We administer the drug to all mice by gavage once daily at around 11:00 a.m. for 60 consecutive days.

### 2.4 Open field test (OFT)

The open field test (OFT) is an experimental behavioral method used to assess the spontaneous locomotor activity, exploratory behavior, and anxiety-like behavior of laboratory animals. The OFT apparatus consists of an observation box and analysis software. The observation box is 30–40 cm in height, with an activity area measuring 300 × 300 mm at the bottom. The inner walls of the observation box are opaque, and the camera system is positioned above the activity area. During the experiment, the activity of mice within the activity area is recorded over a 10 min period. After the experiment, the mouse feces and urine in the activity area are cleaned with 75% alcohol to minimize the influence of information and cues left by the previous mouse on the next experimental mouse. The fully automated social behavior recognition and analysis system (Noldus (Beijing) Information Technology Co. Ltd.) records parameters such as trajectory visualization images, driving distance, and speed.

### 2.5 New object recognition (NOR) experiment

The novel object recognition test is an animal behavior experiment used to assess the memory and cognitive function of laboratory animals, particularly mice and rats. The apparatus for the novel object recognition test is similar to that used in the open field test. Familiarization period: Two identical objects are placed on either side of the experimental chamber, fixed in position such that they are approximately 6 cm from the edge of the observation chamber. The mouse is placed in the observation chamber with its back facing the objects and allowed to freely explore for 5 min. The frequency and duration of the mouse’s interactions with the two objects during this period are recorded. After the test, the mouse is removed and returned to its cage. Urine and feces are cleaned from the observation box, and the experimental box and objects are wiped with 75% alcohol to prevent residual odors from the previous mouse from affecting the next mouse. Testing period: After completing the familiarization period for 1 day, test the mouse. The position of one object in the observation box remains the same as during the familiarization period (A1, old object), while the other object is replaced with a new object (A2, new object). Place the mouse in the observation box with its back facing the objects and allow it to explore for 5 min. Record the time and frequency of the mouse’s contact with the old and new objects within the 5 min period. Clean and disinfect after the test. The fully automated social behavior recognition and analysis system [Noldus (Beijing) Information Technology Co. Ltd.,] recorded the time each mouse spent exploring objects. The mouse’s learning and memory ability was evaluated using the recognition index (RI) = A2/(A2 + A1) × 100%.

### 2.6 Morris water maze (MWM) test

The MWM test is an experimental behavioral method used to study spatial learning and memory abilities. It is widely used in research on the function of the hippocampus in the animal brain and cognitive behavioral disorders (Lissner et al., 2021). It is mainly used to test small laboratory animals, such as mice and rats. The Morris water maze apparatus consists of analysis software, a platform, a water tank, and a video monitoring system. The water tank is circular with a diameter of 120 cm, a water depth of 0.5 m, and a water temperature of approximately 25°C. Food-grade titanium dioxide is added to the water to make it milky white, facilitating observation of black mice. The water tank is divided into four equal quadrants: I, II, III, and IV. A circular platform with a diameter of 6 cm is fixed in Quadrant II, submerged 1 cm below the water surface to remain invisible to the mice. Geometric visual markers are placed along the pool edge to aid spatial cognition. During experiments, a light shield is used to prevent light interference, and experimenters maintain silence. The Morris Water Maze Analysis System (Guangzhou Feidi Biotechnology Co., Ltd.) records swimming trajectories, latency, average speed, total swimming distance, time spent in each quadrant, and the number of platform crossings. During the adaptation phase, each mouse underwent two training sessions. The platform was slightly above the water surface by 1 cm. Mice were randomly placed in the water and allowed to swim freely for 1 min to observe swimming patterns, adapt to the environment, and assess swimming ability. If the mouse found the platform, the escape latency was recorded, and the mouse was allowed to stay on the platform for 20 s. If not, the mouse was guided to the platform and stayed there for 20 s, with the latency recorded as 60 s. Afterward, the mouse was removed, dried, and returned to the cage. The positioning navigation test was conducted for 5 days, three times daily. The platform was hidden 1 cm below the water surface. Mice entered the pool from a randomly selected quadrant and swam for 1 min. If they found the hidden platform, the escape latency was recorded, and they remained on the platform for 5 s. Otherwise, they were guided to the platform and remained there for 10 s to reinforce memory, with the latency recorded as 60 s. The spatial exploration test is conducted on the 6th day. After removing the platform, the mouse is placed in the water from quadrant IV, and the number of times it crosses the original platform area and the dwell time in the quadrant where the original platform was located are recorded within 60 s.

### 2.7 16S rRNA amplicon sequencing

#### 2.7.1 DNA extraction

Fresh fecal samples from each mouse were collected into sterile 1.5 mL EP tubes, snap-frozen on dry ice (−78°C), and stored at −80°C until processing. Genomic DNA was extracted using the MOBIO PowerSoil^®^ DNA Isolation Kit (catalog 12888-100; MOBIO Laboratories, Carlsbad, CA, United States) following the manufacturer’s protocol. DNA purity and concentration were assessed on a Nanodrop One spectrophotometer (Thermo Fisher Scientific, ND-ONE-W).

#### 2.7.2 PCR amplification

The V4 region of the 16S rRNA gene was amplified using barcode-labeled primers 515F and 806R. Each 50 μL reaction contained:

25 μL 2 × Premix Taq (TaKaRa RR902A; Takara Biotechnology, Dalian, China).
1 μL Forward primer (10 μM).
1 μL Reverse primer (10 μM).
50 ng template DNA.
Nuclease-free water to 50 μL.
Thermal cycling (Bio-Rad S1000 PCR System) was:
1. 94°C for 5 min.
2. 30 cycles of: 94°C for 30 s, 52°C for 30 s, 72°C for 30 s.
3. 72°C for 10 min
4. Hold at 4°C

Amplicons were verified on a 1.5% agarose gel (Tanon 4100), yielding a single band of ∼290–310 bp. Band intensities were quantified with GeneTools v4.03 (SynGene) to ensure equal mass pooling.

#### 2.7.3 Sequencing and data processing

Pooled PCR products were purified from gel slices using the E.Z.N.A.^®^ Gel Extraction Kit (catalog D2500-00; Omega Bio-tek, United States), eluted in TE buffer, and quantified by Qubit Broad-Range DNA Assay Kit (catalog Q32853; Invitrogen). Libraries were prepared with the ALFA-SEQ DNA Library Prep Kit (catalog number per provider) following standard end-repair, A-tailing, and adapter-ligation steps. Library size (∼400 bp) was checked on a Qsep400 system (Hangzhou Hooiz), concentration measured on Qubit 4.0, then sequenced on an Illumina MiSeq using 2 × 250 bp paired-end reads.

Raw FASTQ files were quality-trimmed with fastp (v0.14.1) and primers removed by cutadapt. Paired reads were merged (min overlap 16 bp, max mismatch 5 bp) into raw tags, further quality-filtered into clean tags, and clustered into OTUs (97% identity via UPARSE) or ASVs (DADA2). Representative sequences were taxonomically assigned against the SILVA database (confidence ≥ 0.8) using USEARCH SINTAX, with chloroplast/mitochondrial contaminants removed. Downstream analyses included alpha diversity (Chao1, Shannon, Simpson; rarefaction and rank-abundance curves), beta diversity (NMDS, PCA, PCoA), differential abundance (LEfSe, Kruskal–Wallis, Wilcoxon tests), and functional prediction with PICRUSt2 to infer COG and KEGG pathway abundances.

### 2.8 Statistical analysis

All data were obtained from independently prepared samples with at least three replicates. Data were analyzed and plotted using SPSS 26.0 software package (IBM Corporation.) and GraphPad Prism 10.4.0 software package (GraphPad Software Inc.). Data are expressed as mean ± standard deviation (SD). Data were analyzed by one-way ANOVA followed by *LSD* or *Dunnett’s T3* test. Statistical *P* < 0.05 was taken as the level of significance.

## 3 Result

### 3.1 Effects of Danggui Shaoyao San and its disassembled prescriptions on anxiety-like behavior in AD mice

**FIGURE 1:**
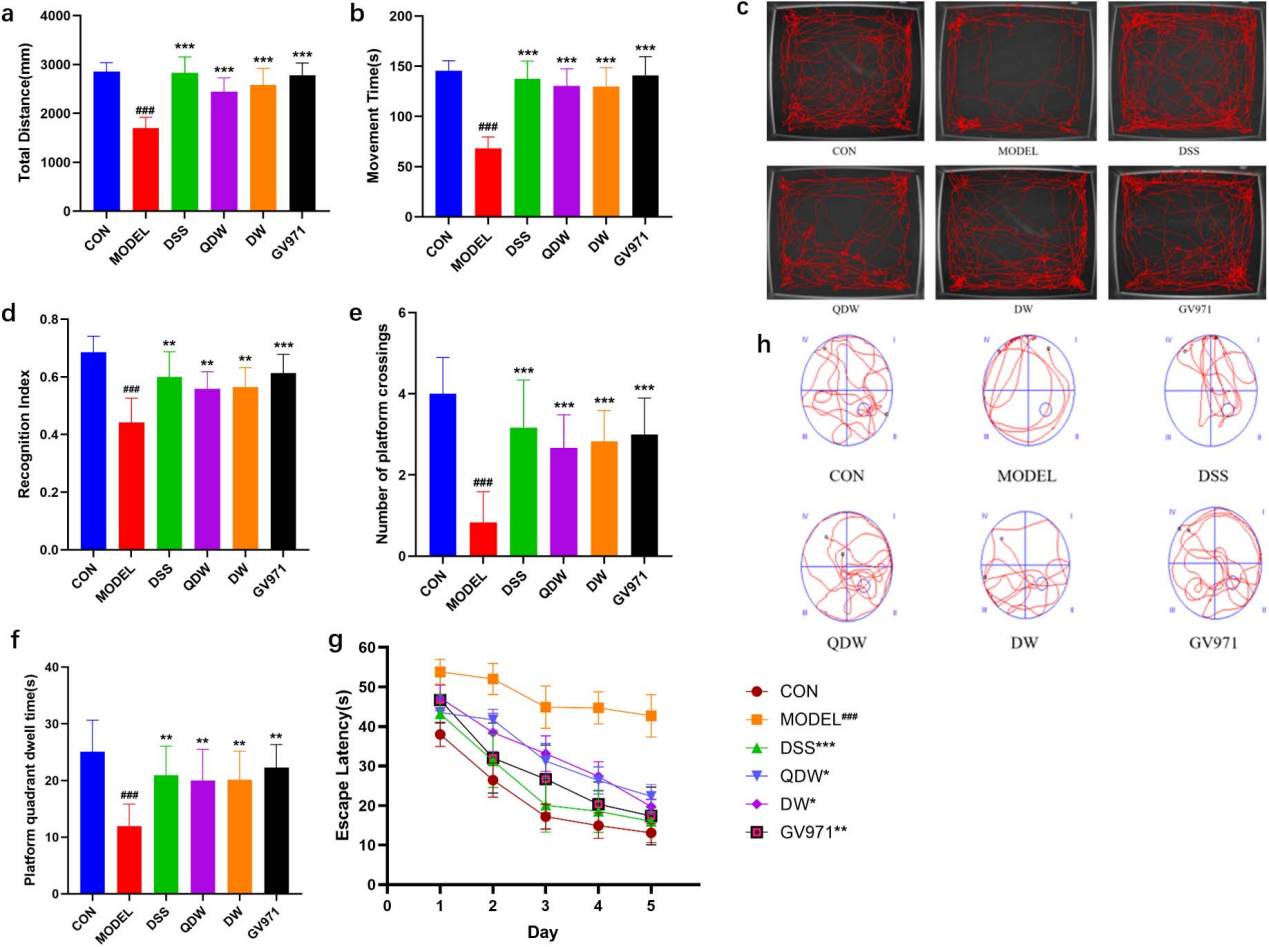
The effects of Danggui Shaoyao San and its disassembled prescriptions on behavioral disorders and cognitive deficits in mice. **(a)** The effect of Danggui Shaoyao San and its disassembled prescriptions on the movement distance of Alzheimer’s disease (AD) mice in the OFT. **(b)** The effect of A Danggui Shaoyao San and its disassembled prescriptions on the activity time of AD mice in the open field test (OFT). **(c)** Representative diagrams of the trajectories of activity in the central area of the open field for each group of mice. **(d)** The effects of Danggui Shaoyao San and its disassembled prescriptions on recognition index (RI) in AD mice. **(e)** The effect of Danggui Shaoyao San and its disassembled prescriptions on the number of crossings of AD mice on the platform. **(f)** The effect of Danggui Shaoyao San and its disassembled prescriptions on the platform quadrant dwell time of AD mice. **(g)** The effects of Danggui Shaoyao San and its disassembled prescriptions on the escape latency in AD mice. **(h)** Representative diagrams of swimming trajectories for each group of mice. *n* = 6, Compared with the control (CON) group, ^###^*P* < 0.001; compared with the MODEL group, *P < 0.05, **P < 0.01, ***P < 0.001.

Figures 1a, b show the open field test results for each group of mice, including total distance and movement time. Compared with the CON group, the MODEL group exhibited a significant decrease in both total distance and movement time (*P* < 0.001). Compared with the MODEL group, the GV971 group also showed a marked reduction in total distance and movement time (*P* < 0.001). Similarly, the DSS, QDW, and DW groups all demonstrated significantly reduced total distance and movement time relative to the MODEL group (*P* < 0.001). Figure 1c presents representative movement trajectories within the center area of the open field for each group, illustrating that the MODEL group mice traveled shorter distances.

### 3.2 Effects of Danggui Shaoyao San and its disassembled prescriptions on cognitive and exploratory abilities in AD mice

Figure 1d shows that two months after treatment, the recognition index in all treatment groups was significantly increased. Compared with the CON group, the MODEL group’s recognition index was significantly decreased (*P* < 0.001); compared with the MODEL group, the GV971 group’s recognition index was significantly increased (*P* < 0.001); and compared with the MODEL group, the DSS, QDW, and DW groups’ recognition indices were all significantly increased (*P* < 0.01).

### 3.3 Effects of Danggui Shaoyao San and its disassembled prescriptions on improvement of spatial learning and memory impairments in AD mice

Figure 1g shows that after five days of training, the escape latency in all groups decreased as training days increased. Compared with the control group, the MODEL group’s escape latency was significantly increased (*P* < 0.001); compared with the MODEL group, the GV971 group’s escape latency was significantly reduced (*P* < 0.01); and compared with the MODEL group, the DSS, QDW, and DW groups’ escape latencies were all significantly reduced (*P* < 0.05). As shown in Figures 1e, f during the spatial exploration phase (with the platform removed), the number of platform crossings and the time spent in the target quadrant were compared among all groups. Compared with the CON group, the MODEL group showed a significant reduction in both platform crossings and time spent in the target quadrant (*P* < 0.001). Compared with the MODEL group, the GV971 group exhibited a significant increase in platform crossings (*P* < 0.001) and a significant increase in time spent in the target quadrant (*P* < 0.01). Similarly, compared with the MODEL group, the DSS, QDW, and DW groups all displayed a significant increase in platform crossings (*P* < 0.001) and a significant increase in time spent in the target quadrant (*P* < 0.01). Figure 1h presents representative swim paths for each group, illustrating that the MODEL group mice had fewer platform crossings and spent less time in the target quadrant.

### 3.4 Analysis of the structure and abundance of gut microbiota

**FIGURE 2:**
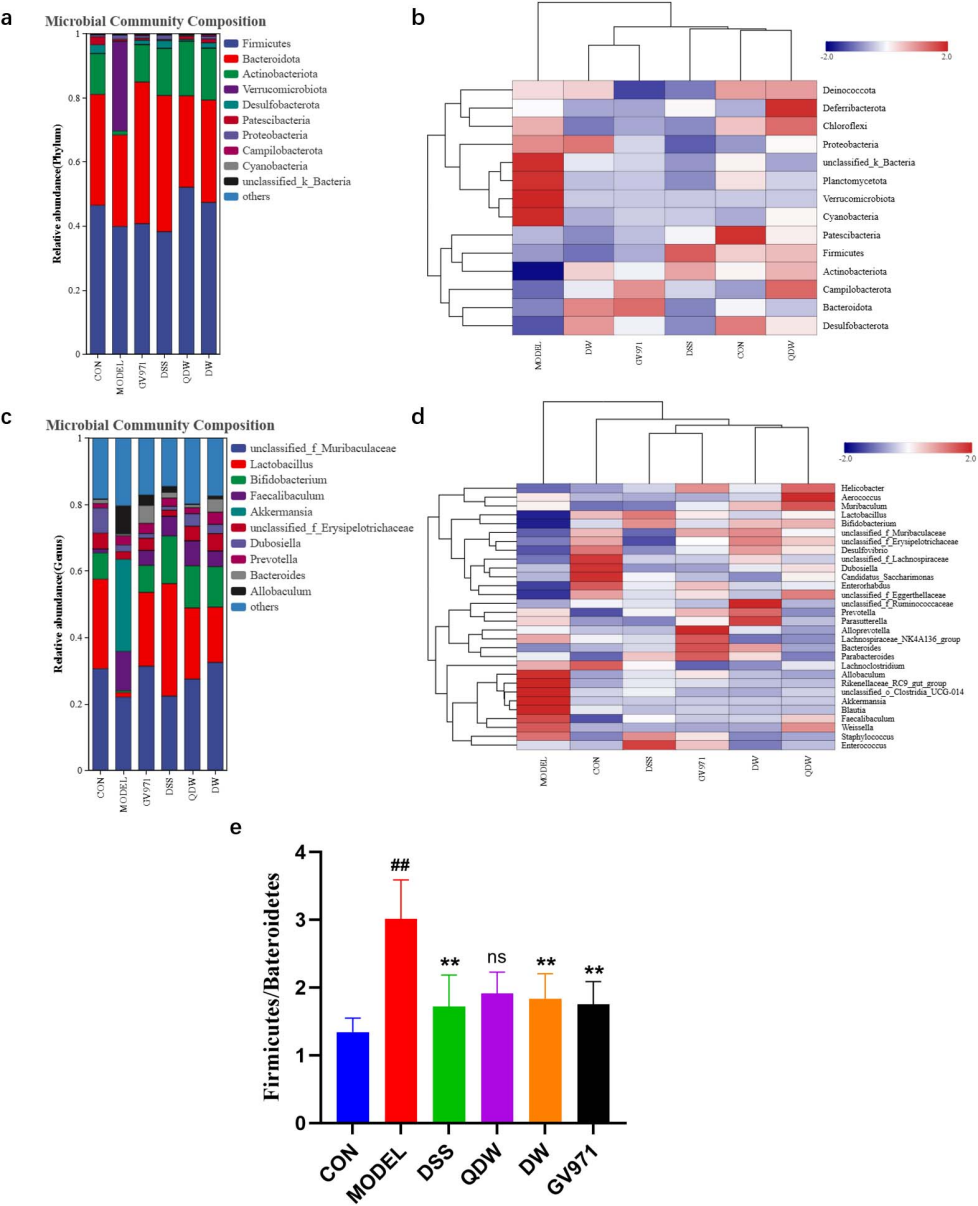
Analysis of the effects of Danggui Shaoyao San and its disassembled prescriptions on the structure of gut microbiota in APP/PS1 mice. **(a)** Histogram of relative abundance at the phylum level of each group under the intervention of Danggui Shaoyao San. **(b)** Heatmap of clustering at the phylum level for each sample. **(c)** Histogram of relative abundance at genus level of each group under the intervention of Danggui Shaoyao San. **(d)** Heat map of clustering at genus level for each sample. **(e)** The valves of Bacteroidota/Firmicutes. Each color in the relative abundance histogram represents a phylum or genus, and the length of the color block represents the abundance ratio of the phylum or genus; the top 10 phylum or genus in terms of abundance are shown, and those with a smaller percentage of abundance are combined as “others.” The clustering heatmap indicates different groupings horizontally and different taxonomic structures of the colony vertically, and the colors indicate the magnitude of species abundance, with darker red indicating higher species abundance and darker blue indicating lower species abundance. *n* = 5 or 6, Compared with the CON group, ^##^*P* < 0.01; compared with the MODEL group, **P < 0.01.

Figure 2a shows the distribution of phylum-level flora structure in each group of mice. Key observations regarding the relative abundance of major phyla include: Verrucomicrobiota: Higher in the MODEL group compared to CON, and a decrease was observed in all treatment groups (DSS, QDW, DW, GV971) relative to MODEL; Actinobacteriota: Increased in the DSS, QDW, DW, and GV971 groups compared to MODEL, with the QDW group showing the highest abundance; Firmicutes: Increased in the QDW and DW groups relative to MODEL, and minimal change was noted in the DSS group; Bacteroidota: Increased in the DSS and DW groups compared to MODEL, and minimal change was noted in the QDW group.

It was also found through the histogram of abundance at the phylum level that the relative abundance of the phylum Firmicutes and Bacteroidetes was higher than the other microbiota at the phylum level. Figure 2e shows that the ratio value of Firmicutes/Bacteroidetes (F/B value) was significantly higher in the MODEL group compared with the CON group (*P* < 0.01); compared with the MODEL group, the F/B value of the GV971 group, as well as the DSS group and the DW group, was significantly lower (*P* < 0.05). The F/B value of the QDW group was also lower, but the difference was not statistically significant (*P* > 0.05).

The structural distribution of the flora at the genus level in each group of mice is shown in Figure 2c At the genus level, *Akkermansia* [a mucin-degrading genus often implicated in gut barrier modulation but also associated with inflammation in certain dysbiotic states (Rodrigues et al., 2022)], *Faecalibaculum*, *Allobaculum*, and *Dubosiella* were increased in the MODEL group compared with the CON group. The relative abundance of *Lactobacuillus* [a genus of beneficial commensals known for producing lactate and short-chain fatty acids (SCFAs), contributing to gut barrier integrity and immune regulation (Wang et al., 2021; Gu et al., 2023)], *Bifidobacterium* [another key beneficial genus involved in carbohydrate fermentation, SCFA production, and immune modulation (Hung et al., 2022)] was elevated in the DSS, QDW, DW and GV971 groups compared to the MODEL group, with the highest in the DSS group; and *Bacteroides* [a dominant genus crucial for polysaccharide degradation and SCFA production (Liu et al., 2022)] in the DSS, QDW, DW groups, *unclassified_f_Muribaculaceae* [a family containing important mucin and glycan degraders also involved in SCFA production (Zhu et al., 2024)] in DSS, QDW and DW groups were all elevated in relative abundance compared to the MODEL group, and there was a decrease in the abundance of *Akkermansia*, *Faecalibaculum*, *Allobaculum*, and *Dubosiella*.

After the intervention of Danggui Shaoyao San, the clustering heat map of each group of mice at the phylum level was shown in Figure 2b The structure of the bacterial flora of mice in the CON group was significantly different from that of the model group, and the abundance of Patescibacteria in the mice in the CON group was higher. The relative abundance of Plantomycetota, Verrucomicrobiota, and Cyanobacteria was higher in mice in the MODEL group compared to mice in the CON group. After the administration of drug intervention, the bacterial structure of mice was significantly improved, and the relative abundance of Firmicutes increased the most in the DSS group compared to the MODEL group, the relative abundance of Deferribacteria was significantly up-regulated in the QDW group, and the aggregation of Proteobacteria was increased in the DW group. The relative abundance of Bateroidota was higher in the GV971 group than in the other groups. Overall, the types of phylum in the gut microbiota of mice remained relatively unchanged after the drug intervention, but the abundance showed significant changes, with different groupings of Danggui Shaoyao San affecting different phylums.

The results of the heat map analysis of species abundance clustering in Figure 2d indicated the differences in species abundance clustering at the genus level in each group of mice after the intervention of Danggui Shaoyao San samples. The structure of the bacterial colony in the CON group was significantly different from that of the MODEL group, which showed higher relative abundance of *Allobaculum*, *Akkermansia*, Blautia, and *Clostridia_UCG-014* in higher relative abundance. Compared with the MODEL group, the genus level changed after the drug intervention, the relative abundance of *Enterococcus* in the DSS group was higher than the other groups, the relative abundance of *Aerococcus* was the highest in the QDW group, and the relative abundance of *Ruminococcaceae* in the DW group was higher than the other groups. The relative abundance of *Alloprovotella* was highest in the GV971 group. In summary, the gut microbiota structure of mice at the genus level changed significantly in composition and abundance after the pharmaceutical intervention of Danggui Shaoyao San.

### 3.5 Alpha diversity Analysis

**FIGURE 3:**
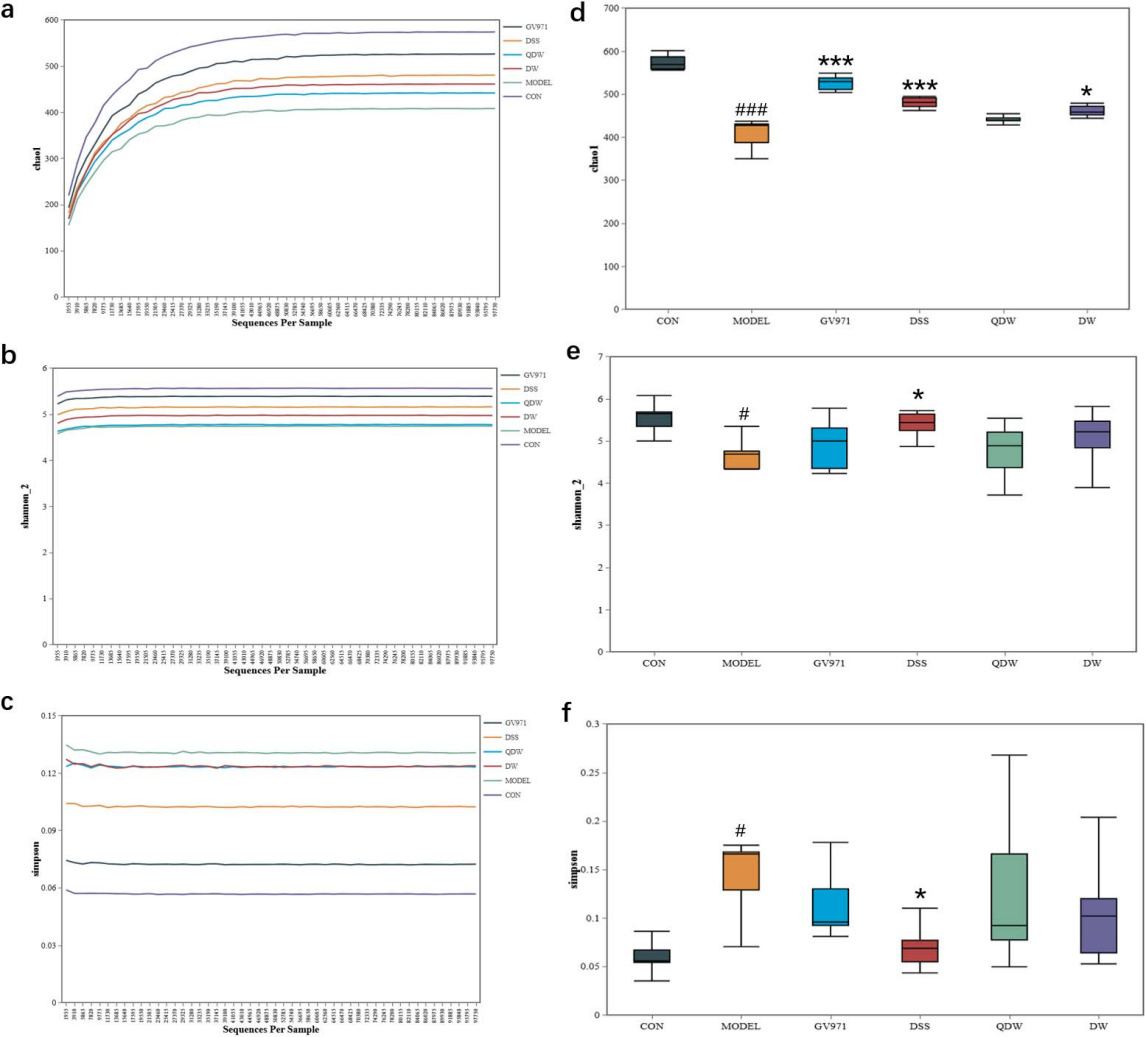
Effects of Danggui Shaoyao San and its disassembled prescriptions on the Alpha diversity of gut microbiota in APP/PS1 mice. **(a)** Chao1 rarefaction curve; **(b)** Shannon rarefaction curve; **(c)** Simpson rarefaction curve; **(d)** Chao1 index; **(e)** Shannon index; **(f)** Simpson index. Compared with the control (CON) group, ^#^*P* < 0.05 and ^###^*P* < 0.001; compared with the MODEL group, *P < 0.05 and ***P < 0.001.

The Chao1, Shannon, and Simpson rarefaction curves of the gut microbiota in each group of mice are shown in Figures 3a–c. These curves illustrate the microbial diversity at different sequencing depths for various drug intervention groups. Specifically, the Chao1 and Shannon curves are positively correlated with species diversity—that is, a higher index indicates greater species diversity—whereas the Simpson curve is inversely correlated with species diversity, meaning a higher index indicates lower species diversity. The results show that mice in the MODEL group had the lowest Chao1 and Shannon indices and the highest Simpson index, indicating that species diversity was the lowest in the MODEL group. After intervention with Danggui Shaoyao San, compared with the MODEL group, species diversity increased in all treatment groups. Among these, the DSS group exhibited the highest species diversity, most closely approximating that of the CON group, though still lower than that of the GV971 group.

Figures 3d–f show the Chao1, Shannon, and Simpson indices of the gut microbiota in the various treatment groups of mice. The Chao1 index, which reflects the species richness of the microbial community, was significantly decreased in the MODEL group compared with the CON group (*P* < 0.001); compared with the MODEL group, the Chao1 index increased in the DSS, QDW, DW, and GV971 groups, with statistically significant differences observed in the GV971 (*P* < 0.001), DSS (*P* < 0.001), and DW (*P* < 0.05) groups. The Shannon index, which reflects species diversity, was significantly lower in the MODEL group than in the CON group (*P* < 0.05); compared with the MODEL group, the Shannon index increased in the GV971, DSS, QDW, and DW groups, with the increase in the DSS group being statistically significant (*P* < 0.05). The Simpson index, which reflects the evenness of the microbial community, was significantly higher in the MODEL group compared with the CON group (*P* < 0.05); compared with the MODEL group, the Simpson index decreased in the GV971, DSS, QDW, and DW groups, with the difference in the DSS group reaching statistical significance (*P* < 0.05). Collectively, the significantly lower Chao1 and Shannon indices and higher Simpson index observed in the MODEL group indicate a state of reduced microbial diversity and ecological imbalance (dysbiosis), characteristic of AD pathology. Conversely, the elevation of Chao1 and Shannon indices and reduction of the Simpson index in the DSS, QDW, DW, and GV971 groups, particularly the pronounced effect in the DSS group, suggest that these treatments restored a more diverse, balanced, and ecologically resilient gut microbiota community.

### 3.6 Beta diversity analysis

**FIGURE 4:**
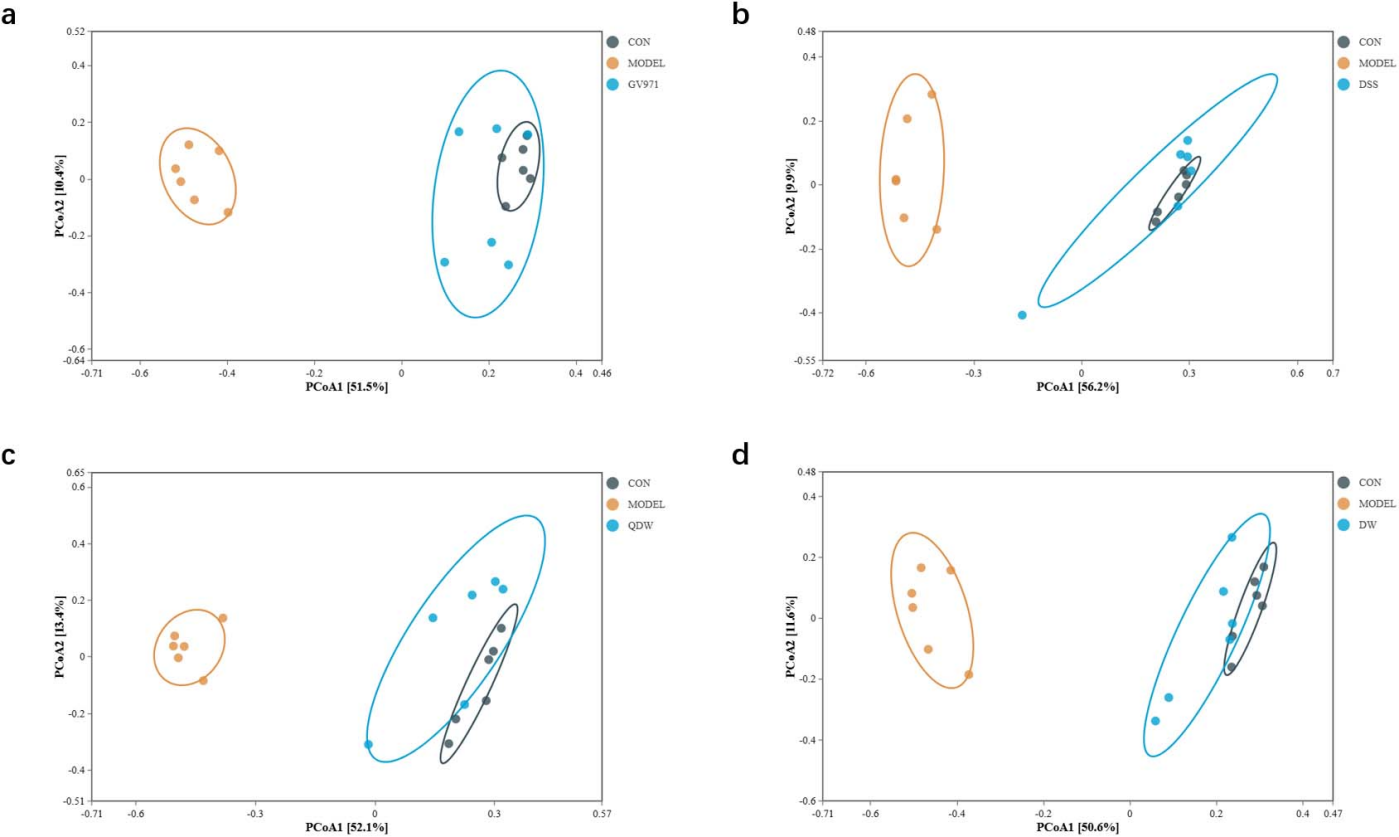
PCoA analysis of the gut microbiota in each group of mice following administration of Danggui Shaoyao San and its disassembled prescriptions. **(a)** Inter-group analysis of the gut microbiota between the control (CON) and MODEL groups and the GV971 group. **(b)** Inter-group analysis of the gut microbiota between the CON and MODEL groups and the Danggui Shaoyao San (DSS) group. **(c)** Inter-group analysis of the gut microbiota between the CON and MODEL groups and the QDW group. **(d)** Inter-group analysis of the gut microbiota between the CON and MODEL groups and the DW group.

Figures 4a–d represent the inter-group analysis of the gut microbiota between the CON and MODEL groups and the GV971, DSS, QDW, and DW groups, respectively. The contribution rates of PCoA1 were 51.5%, 56.2%, 52.1%, and 50.6%, while those of PCoA2 were 10.4%, 9.9%, 13.4%, and 11.6%. Compared with the CON and MODEL groups, the overall contribution rate was highest in the DSS group at 66.1%. The gut microbiota distributions of the GV971, DSS, QDW, and DW groups all overlapped with that of the CON group, with the DSS treatment brought the microbiota composition closest to that of healthy controls. Anosim analysis presented in Table 1 showed that the inter-group R values for the GV971, DSS, QDW, and DW groups compared with the CON and MODEL groups were 0.728, 0.677, 0.749, and 0.712, respectively, indicating that inter-group differences were greater than intra-group differences and that these differences were statistically significant (*P* = 0.001).

**TABLE 1:** Anosim analysis of gut microbiota in various groups of mice.

Group 1	Group 2	Group 3	R	P
CON	MODEL	GV971	0.728	0.001
CON	MODEL	DSS	0.677	0.001
CON	MODEL	QDW	0.749	0.001
CON	MODEL	DW	0.712	0.001

### 3.7 Linear discriminant analysis effect size (LEfSe)

**FIGURE 5:**
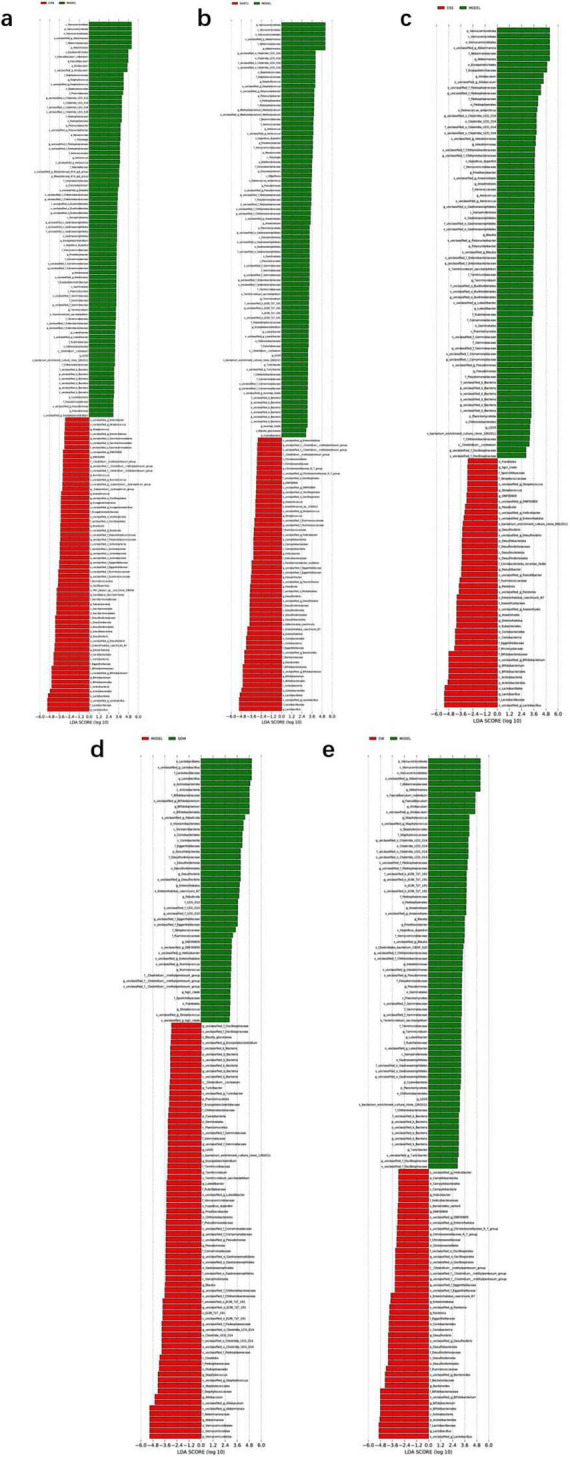
LDA plots of key microbiota comparing different groups with the MODEL group. **(a)** The linear discriminant analysis effect size (LEfSe) analysis comparing the control (CON) group and the MODEL group; **(b)** The LEfSe analysis comparing the GV971 group and the MODEL group; **(c)** The LEfSe analysis comparing the Danggui Shaoyao San (DSS) group and the MODEL group. **(d)** The LEfSe analysis comparing the QDW group and the MODEL group. **(e)** The LEfSe analysis comparing the DW group and the MODEL group. Two-by-two comparisons between all groups were made using a threshold of 2, indicating significance *P* = 0.05.

According to the results of Figure 5a, compared with the CON group, the MODEL group exhibited increased abundance of Verrucomicrobiota, Pseudomonadaceae, Clostridia, *Staphylococcus*, the Clostridiales UCG-014 subgroup, Erysipelotrichales, Akkermansiaceae, Phormidiaceae, *Allobaculum*, *Pseudomonas*, and *Aerococcus*, while the abundance of Firmicutes, Actinobacteria, Saccharimonadia, Desulfovibrionia, Lactobacillales, Bifidobacteriales, *Desulfovibrio*, *Ruminococcus*, *Candidatus_Saccharimonas*, and *Streptococcus* was decreased.

According to the results of Figure 5b, compared with the MODEL group, the GV971 group showed increased abundance of Actinobacteria, Desulfobacteriota, Bacteroidia, Bifidobacteriales, Lactobacillales, *Enterorhabdus*, *Flavonifractor*, *Streptococcus*, *Anaerostipes*, *Enterorhabdus* caecimuris_B7, Adlercreutzia caecimuris, Eggerthellaceae, and Parahymenobacter, while the abundance of Verrucomicrobiota, *Staphylococcus*, the Clostridiales UCG-014 subgroup, Akkermansiaceae, *Polynucleobacter*, Terrimicrobium sacchariphilum, and Deinococcus antarcticus decreased.

According to the results in Figures 5c–e comparing DSS and its disassembled prescription groups with the MODEL group, both the DSS and disassembled groups increased the abundance of Actinobacteria, Bifidobacteriales, Lactobacillaceae, *Desulfovibrio*, and s_Enterorhabdus_caecimuris_B7, while decreasing the abundance of Verrucomicrobiota, Pseudomonadaceae, Akkermansiaceae, *Allobaculum*, Blautia, Akk, and s_Hypsibius_dujardini. Specifically, the DSS group further increased the abundance of Eubacteriales, Microcystaceae, Anaerofustaceae, *Enterorhabdus*, *Anaerostipes*, *Ralstonia*, and *Raoultibacter* while reducing that of Erysipelotrichales; the QDW group increased the abundance of Vicinamibacteria, Streptococcaceae, *Paludicola*, and *Ruminococcus*; and the DW group increased the abundance of Desulfovibrionia, Christensenellaceae, *Bacteroides*, *Ralstonia*, *g_Christensenellaceae_R_7_group*, and s_Bacteroides_sartorii, while decreasing the abundance of s_Faecalibaculum_rodentium and s_Terrimicrobium_sacchariphilum.

### 3.8 16S functional prediction

**FIGURE 6:**
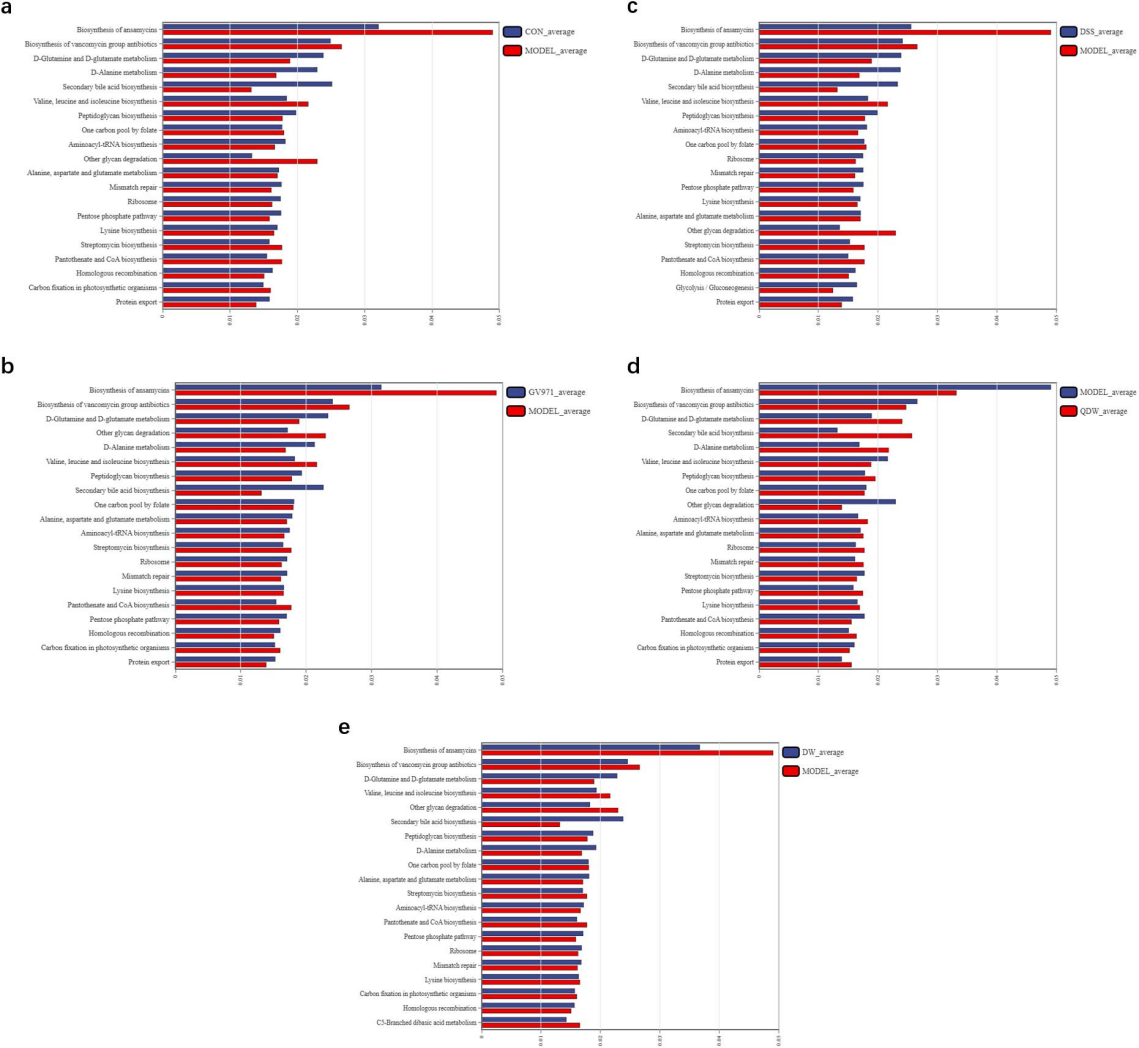
16S functional prediction analysis of the effect of Danggui Shaoyao San and its disassembled prescriptions on the gut microbiota of APP/PS1 mice. **(a)** The comparative analysis of pathway differences between the control (CON) and MODEL groups. **(b)** The comparative analysis of pathway differences between the MODEL and GV971 groups. **(c)** The comparative analysis of pathway differences between the MODEL and DSS groups. **(d)** The comparative analysis of pathway differences between the MODEL and QDW groups. **(e)** The comparative analysis of pathway differences between the MODEL and DW groups.

In this study, we selected L3-level KEGG pathways to perform comparative pathway analysis between groups to elucidate the potential mechanisms underlying the therapeutic effects of DSS and its disassembled prescriptions. In Figure 6a, compared with the CON group, the MODEL group was enriched in several metabolic pathways, including the biosynthesis of ansamycins, the biosynthesis of vancomycin group antibiotics, streptomycin biosynthesis, pantothenate and CoA biosynthesis, carbon fixation in photosynthetic organisms, and the biosynthesis of valine, leucine, and isoleucine. In Figure 6b, compared with the MODEL group, the GV971 group exhibited higher enrichment in pathways such as D-glutamine and D-glutamate metabolism, D-alanine metabolism, secondary bile acid biosynthesis, peptidoglycan biosynthesis, ribosome, mismatch repair, the pentose phosphate pathway, homologous recombination, protein export, and alanine, aspartate and glutamate metabolism. In Figures 6c–e, after intervention with DSS and its disassembled prescriptions, the altered pathways in the gut microbiota of the APP/PS1 mice were largely consistent across groups. Compared with the MODEL group, ten pathways showed increased enrichment, including D-glutamine and D-glutamate metabolism, secondary bile acid biosynthesis, D-alanine metabolism, aminoacyl-tRNA biosynthesis, ribosome, mismatch repair, the pentose phosphate pathway, homologous recombination, and alanine, aspartate and glutamate metabolism, while six pathways exhibited decreased enrichment, namely the biosynthesis of ansamycins, the biosynthesis of vancomycin group antibiotics, one carbon pool by folate, streptomycin biosynthesis, other glycan degradation, and pantothenate and CoA biosynthesis. Additionally, the DSS group was also involved in the enrichment of pathways related to the biosynthesis of valine, leucine, and isoleucine as well as glycolysis/gluconeogenesis, whereas the QDW and DW groups showed decreased enrichment in the carbon fixation pathway in photosynthetic organisms. Both the DSS and QDW groups exhibited increased enrichment in pathways related to protein export and lysine biosynthesis. These results indicate that DSS and its disassembled prescriptions improve metabolic pathways associated with amino acid metabolism, carbohydrate metabolism, fatty acid metabolism, and secondary metabolite synthesis, as well as protein and nucleic acid metabolism.

## 4 Discussion

APP/PS1 mice are a well-established animal model for studying Alzheimer’s disease; by introducing human APP and PS1 mutant genes, these mice produce excessive amyloid-β (Aβ), particularly the highly toxic Aβ_42_, which accumulates in brain tissue to form amyloid plaques and faithfully reproduces the early pathological features of AD. As the mice age, Aβ deposition progressively worsens, and the pathology extends from initial Aβ accumulation to synaptic and neuronal dysfunction, providing a comprehensive window for observing the dynamic progression of AD. Behaviorally, APP/PS1 mice exhibit declines in learning ability, memory impairment, and increased anxiety—cognitive and emotional disturbances that closely mirror those seen in AD patients. Because of these similarities, APP/PS1 mice are widely used in AD research as a primary choice for investigating Aβ-related pathological mechanisms, testing novel therapeutics, and developing potential treatment strategies (Jankowsky et al., 2001; Radde et al., 2006; Ameen-Ali et al., 2017).

Danggui Shaoyao San (DSS) originates from Zhang Zhongjing’s “The Synopsis of the Golden Chamber” and is known for promoting blood circulation, unblocking the meridians, soothing the liver, and regulating the spleen. It was primarily used to treat gynecological diseases. However, as research on traditional Chinese medicine formulas has advanced, many scholars have discovered that DSS can be used to treat AD and improve cognitive deficits in AD patients, thereby enhancing their quality of life. From a traditional Chinese medicine perspective, AD falls under the category of dementia, and its pathogenesis mainly involves dysfunction of the internal organs, insufficient qi and blood, phlegm turbidity (potentially reflecting pathological accumulation like amyloid-beta plaques and neuroinflammation) obstructing the orifices, and blood stasis (possibly indicating microvascular dysfunction and impaired cerebral perfusion) blocking the channels. Through multiple mechanisms such as promoting blood circulation and removing blood stasis, supplementing and nourishing the blood, strengthening the spleen and eliminating dampness, and harmonizing the liver and spleen, DSS can help improve cerebral blood supply, nutritional support, and emotional state in AD patients. This, in turn, alleviates pathological factors such as insufficient cerebral qi and blood, phlegm turbidity obstructing the orifices, and blood stasis, thereby improving memory and cognitive function to some extent.

In recent years, the relationship between the gut microbiota and human health has attracted increasing attention. Alterations in the gut microbiota can affect the host’s immune system and trigger diseases (Zheng et al., 2020). Studies have shown that patients with inflammatory bowel disease, allergies, and neurological disorders exhibit significant changes in the composition of their gut microbiota (Ruigrok et al., 2023). Therefore, investigating the composition, function, and metabolic changes of the gut microbiota is essential to clarify its role in the onset and progression of AD, which holds practical significance for developing new prevention and treatment strategies. Increasing evidence indicates that a bidirectional communication system exists between the brain and the gastrointestinal tract, with the gut microbiota playing a crucial role. This network, known as the gut-brain axis, links the gastrointestinal system with the central nervous system (Liu et al., 2015). Numerous studies have demonstrated that dysbiosis in the gut microbiota can lead to increased permeability of both the intestinal barrier and the blood-brain barrier, potentially mediating or influencing the pathogenesis of AD and other neurodegenerative diseases, especially those associated with aging. The gut microbiota affects the production of metabolic products, pro-inflammatory factors, and neurotransmitters such as short-chain fatty acids (SCFAs), lipopolysaccharide (LPS), gamma-aminobutyric acid (GABA), and 5-hydroxytryptamine (5-HT), thereby improving cognitive deficits caused by AD (Das and Ganesh, 2023). SCFAs regulate immune responses, reduce neuroinflammation, enhance the gut barrier, and may cross the BBB to exert neuroprotective effects and promote neuronal health (Doifode et al., 2021; Xie et al., 2023). In addition, Dysbiosis can promote the release of pro-inflammatory cytokines (e.g., IL-1β, IL-6, TNF-α) from gut immune cells into the circulation. These cytokines can signal to the brain via neural and humoral pathways, activating microglia and contributing to neuroinflammation and neuronal damage, hallmarks of AD (Zheng et al., 2020; Chandra et al., 2023). The results of this study indicate that DSS and its disassembled prescriptions can alter the composition and function of the gut microbiota in APP/PS1 mice, making it more similar to that of normal mice. This suggests that the cognitive improvements observed in APP/PS1 mice treated with DSS and its disassembled prescriptions may be partially mediated by changes in the gut microbiota.

In this study’s behavioral experiments, we first used the open field test to assess how Danggui Shaoyao San and its disassembled prescriptions affect anxiety in APP/PS1 mice. Compared with age-matched control mice, APP/PS1 mice in the MODEL group exhibited reduced distance traveled and time spent in the central area of the open field. In contrast, both the DSS group and the disassembled prescription groups increased total distance and movement time compared with the MODEL group. APP/PS1 mice display anxiety-like behavior, which is generally considered a depressive tendency induced by underlying AD pathology; in an unfamiliar environment, anxious APP/PS1 mice tend to stay in the corners, so their level of activity in the central area can reflect both the severity of their emotional disturbance and any improvement (Johansson et al., 2020). The open field test results indicate that Danggui Shaoyao San and its disassembled prescriptions alleviate anxiety-like behavior in APP/PS1 mice to varying extents, with the full-formula group showing the most pronounced effect. Next, we used the novel object recognition test to evaluate each group’s ability to explore and remember new objects. Normal mice typically form memories of objects in the environment and show curiosity toward novel objects. Therefore, by calculating the recognition index (RI)—the proportion of time spent exploring the novel object relative to the total exploration time—we assessed a mouse’s spatial memory and exploratory drive. Compared with the CON group, RI values were reduced in the MODEL group, indicating lower exploratory drive, whereas both the DSS group and the disassembled prescription groups showed increased RI values relative to the MODEL group, with the DSS group exhibiting the most significant improvement. These results demonstrate that DSS and its disassembled prescriptions can enhance spatial memory and exploratory behavior in mice to varying degrees, with the DSS group showing the most pronounced effect. And finally in the Morris water maze test, we evaluated the effects of DSS and its disassembled prescriptions on spatial learning and memory deficits in APP/PS1 mice. APP/PS1 mice exhibit impairments in spatial memory, as evidenced by longer escape latencies to locate the submerged platform and difficulty remembering its location. Compared with age-matched control mice, the MODEL group displayed significantly prolonged escape latencies and fewer platform crossings during the spatial exploration test period. By contrast, both the DSS group and the disassembled prescription groups showed reduced escape latencies and increased platform crossings relative to the MODEL group, indicating improved spatial learning and memory. Notably, the DSS group exhibited the most pronounced improvements. These results suggest that DSS and its disassembled prescriptions can ameliorate spatial learning and memory impairments in APP/PS1 mice, with the DSS group demonstrating the strongest effect.

Firmicutes and Bacteroidota together constitute the major component of the gut microbiota and form the cornerstone of its structure. The ratio of Firmicutes to Bacteroidota is known as the F/B ratio. Bacteroidota are renowned for their complex polysaccharide metabolism; they can break down dietary polysaccharides, cellulose, and mucin from the host’s intestinal mucus layer, releasing short-chain fatty acids (SCFAs) (Pan et al., 2023). Bacteroidota play a crucial role in regulating the immune system and inflammation, and a reduction in their abundance may disrupt the balance of the gut microbiota, leading to altered immune responses and increased systemic inflammation that can result in neuroinflammation and neuronal damage (Vogt et al., 2017). Firmicutes are named for their thick peptidoglycan cell walls; this phylum comprises bacteria with diverse morphologies and metabolic characteristics that are involved in key functions related to gut health and metabolism (Chen et al., 2023). However, the F/B ratio in AD patients is often abnormal. An elevated F/B ratio may reflect an overgrowth of Firmicutes, which can lead to an imbalance in SCFA metabolism; in particular, a decrease in butyrate may weaken the intestinal barrier and increase the risk of toxins such as LPS entering the bloodstream, thereby triggering systemic and neuroinflammation (Minamisawa et al., 2021). Thus, modulating the composition of the gut microbiota to restore a normal F/B ratio may offer new intervention strategies for the prevention and treatment of AD. Zhang’s findings also indicate that GuanXinNing tablet can significantly inhibit oxidative stress and neuronal apoptosis, alleviate AD cognitive impairments, and improve gut microbiota composition while reducing the F/B ratio (Zhang et al., 2021). Cao’s research shows that anthocyanins can alter the gut microbiota composition of mice with cognitive deficits, lower the F/B ratio, and exert neuroprotective regulatory effects (Cao et al., 2024). In this study, we found that the F/B ratio in the gut microbiota of APP/PS1 mice in the MODEL group was higher than that in the CON group. Following treatment with DSS and its disassembled prescriptions, the F/B ratio was significantly reduced, with the DSS group showing the lowest F/B ratio, followed by the DW group and then the QDW group. In this study, we found that the F/B ratio in the gut microbiota of APP/PS1 mice in the MODEL group was higher than that in the CON group. Following treatment with DSS and its disassembled prescriptions, the F/B ratio was significantly reduced, with the DSS group showing the lowest F/B ratio, followed by the DW group and then the QDW group.

This study focused on the effects of DSS and its disassembled prescriptions on the gut microbiota of APP/PS1 mice. Comprehensive analysis of the microbial community structure and LEfSe results indicated that treatment with DSS and its disassembled prescriptions significantly altered the gut microbiota in APP/PS1 mice. After treatment, the abundance of harmful bacteria—including Firmicutes, Verrucomicrobiota, *Akkermansia*, *Pseudomonas*, Blautia, and *Allobaculum*—was reduced, while the abundance of beneficial bacteria such as Bacteroidota, Actinobacteria, *Bifidobacterium*, and Lactobacillus was increased. In addition, the DSS group further increased the abundance of Coriobacteriia, Eubacteriales, Enterorhabdus, *Anaerostipes*, *Ralstonia*, and *Raoultibacter*; in the QDW group, the abundance of Vicinamibacteria, Streptococcaceae, *Paludicola*, and *Ruminococcus* was elevated; and in the DW group, the abundance of the class Desulfovibrionia, Christensenellaceae, and s_Bacteroides_sartorii increased. Notably, the gut microbiota structure of the DSS group was most similar to that of the CON group, suggesting that DSS and its disassembled prescriptions may help restore gut microbiota balance by promoting beneficial bacteria and reducing the relative abundance of pathogenic bacteria, thereby improving cognitive deficits in APP/PS1 mice.

In this study, we compared the differences in microbiota composition within and between groups using diversity analysis. The onset of AD causes dysbiosis and a reduction in gut microbiota diversity, resulting in a marked difference from the microbiota of normal mice. In the alpha diversity analysis, the MODEL group showed lower Chao1 and Shannon indices and a higher Simpson index, indicating that AD reduces microbial diversity. Administration of DSS and its disassembled prescriptions reversed these changes by increasing the Chao1 and Shannon indices and decreasing the Simpson index, thereby enhancing overall diversity. Among the treatment groups, the DSS group exhibited the most significant improvement, followed by the DW group and then the QDW group. In the beta diversity analysis, PCoA revealed a clear difference in microbiota distribution between the MODEL and CON groups. Treatment with DSS and its disassembled prescriptions significantly improved the gut microbiota structure in AD mice, making it more similar to that of the CON group. In this case, the DSS group was most similar to the CON group, followed by the QDW group and finally the DW group. These results indicate that AD alters both the composition and diversity of the gut microbiota, while treatment with DSS and its disassembled prescriptions can significantly enhance species diversity and richness in APP/PS1 mice.

Studies have found that DSS exerts significant therapeutic effects on neurodegenerative diseases through a multi-component, multi-target, and multi-pathway approach. These diseases involve complex interactions among various pathological mechanisms and factors (Wu et al., 2020; Huang et al., 2021; Zhou et al., 2021; Mahaman et al., 2023). This chapter explores the reasons behind the differences in the effects of DSS and its component formulations on gut microbiota, which may be due to variations in the synergistic effects of the herbal combinations. The full formula, by combining multiple herbs, may activate complementary pathways for regulating the microbiota. In contrast, the component formulations may lack key ingredients and thus have a more limited effect on the gut microbiota. The full formula of DSS, through the synergy of its multiple components, may regulate gut microbiota structure more comprehensively, while the component formulations, targeting specific bacterial branches, lack the capacity for systemic regulation, resulting in a weaker modulatory effect on gut microbiota structure.

16S functional prediction analysis revealed that after administration of DSS and its disassembled prescriptions, the microbial communities in APP/PS1 mice may have undergone significant changes in functions related to amino acid metabolism, carbohydrate metabolism, nucleic acid metabolism, fatty acid metabolism, and secondary metabolite synthesis. Among these functions, amino acid metabolism—including the metabolism of glutamine, glutamate, and aspartate—is closely related to neurotransmitter balance. Abnormal accumulation of glutamate can trigger excitotoxicity and lead to neuronal damage, while dysregulation of glutamine metabolism may exacerbate oxidative stress and neuroinflammation, thereby contributing to the development of AD (Chang et al., 2020). In addition, secondary bile acid metabolism plays an important role in regulating both the gut microbiota and the host immune system. Studies have shown that secondary bile acids can modulate inflammatory responses and immune function through interactions with nuclear receptors. Disruption of secondary bile acid metabolism may worsen neuroinflammation via the gut-brain axis, thereby influencing the progression of AD (Nabizadeh et al., 2024). Carbohydrate metabolism pathways, such as the pentose phosphate pathway, maintain cellular antioxidant capacity by generating NADPH, and insufficient NADPH may exacerbate oxidative stress-induced neuronal damage, which is a key factor in neuronal dysfunction and death in AD (Johnson, 2020). Moreover, pathways involved in peptidoglycan synthesis and carbohydrate degradation are linked to microbial metabolites, which may activate the host immune system and induce systemic inflammatory responses, thereby promoting neuroinflammation (Navalón-Monllor et al., 2023; Peng et al., 2023). Therefore, our study suggests that DSS and its disassembled prescriptions may improve cognitive deficits in AD by modulating metabolic pathways related to amino acid metabolism, secondary bile acid metabolism, carbohydrate metabolism, and sugar metabolism.

This study also has limitations, as the precise mechanisms by which DSS and its disassembled prescriptions improve AD pathology in this model are not yet fully elucidated. Future studies should integrate brain tissue analysis to directly link DSS-induced microbial shifts to Aβ/tau pathology and neuroinflammation markers. While our data demonstrate correlative improvements in gut microbiota and cognition, future work employing fecal microbiota transplantation (FMT) or gnotobiotic models could establish causality between specific bacterial alterations and molecular pathways in AD pathogenesis.

While our KEGG-based functional prediction provides valuable insights into microbial metabolic shifts, we acknowledge the inherent limitations of this inference approach in capturing precise protein-level dynamics. In this context, the recent metaproteomic study by Ayan et al.’s (2023) research offer critical experimental validation. Their work in 5xFAD mice demonstrated significant alterations in key microbial proteins including: ATP synthase subunits (decreased in AD models), aligning with our observed enrichment in energy metabolism pathways (e.g., D-glutamine/D-glutamate metabolism); RNA polymerase subunits (increased in AD), correlating with our pathway predictions involving transcription regulation; Chaperonin GroEL (elevated), consistent with our findings of protein conformational pathway modulation. While APP/PS1 and 5xFAD models exhibit temporal differences in amyloid pathology, both recapitulate gut-brain axis dysregulation characteristic of AD progression and share phenotypes of cognitive decline and neuroinflammation (Upadhyay et al., 2024). Notably, both studies converge on dysregulated microbial energy metabolism and protein processing as hallmarks of AD-associated dysbiosis. The metaproteomic evidence of ATP synthase suppression (Ayan et al., 2023) experimentally substantiates our predicted enrichment in ATP pathways (D-Glutamine and D-glutamate metabolism). This complementary evidence strengthens the hypothesis that gut microbiota in AD models exhibits impaired bioenergetic capacity, potentially contributing to systemic metabolic disturbances. Methodologically, our PICRUSt2-based functional prediction and Ayan et al.’s (2023) direct metaproteomic analysis represent complementary approaches. While 16S functional inference provides pathway-level hypotheses with high coverage, metaproteomics offers superior accuracy in quantifying protein expression. The convergence of our KEGG predictions (e.g., amino acid metabolism, secondary bile acid biosynthesis) with their experimentally identified protein markers (ATP synthase, RNA polymerase) suggests that despite methodological differences, core microbial functional disturbances in AD models are robustly detectable. Future studies integrating multi-omics approaches will be essential to dissect causal mechanisms.

In addition, while our study demonstrates that DSS and its disassembled prescriptions ameliorate Alzheimer’s disease-associated cognitive deficits and gut dysbiosis in APP/PS1 mice, we acknowledge a critical limitation highlighted by recent metaproteomic evidence. Ayan et al.’s (2023) findings revealed distinct gut microbiota profiles between 12 months-old healthy aging mice and age-matched AD transgenic mice, indicating that dysbiosis in AD models represents pathology beyond normal aging effects. In our experimental design, the MODEL group (APP/PS1 mice) was compared primarily to young healthy controls (CON group: C57BL/6 mice), potentially conflating age-related microbial shifts with AD-specific alterations. The microbiota changes we observed in APP/PS1 mice—including increased Firmicutes/Bacteroidetes ratio, reduced Lactobacillus and Bifidobacterium abundance, and elevated Akkermansia—nevertheless align with established AD dysbiosis signatures in both human patients and aged AD models (Vogt et al., 2017; Heravi et al., 2023; Wang et al., 2024). Crucially, DSS intervention reversed these AD-associated shifts and restored microbial diversity toward healthy aged states, evidenced by normalized Chao1/Shannon indices approaching CON levels (Figures 3d–f) and PCoA clustering showing DSS-treated microbiota diverging from MODEL while approaching CON (Figure 4b). This suggests DSS selectively counteracts pathology-driven dysbiosis rather than generalized aging effects. Future studies incorporating age-matched healthy controls will be essential to conclusively dissect age-related versus AD-specific contributions.

## 5 Conclusion

Danggui Shaoyao San and its disassembled prescriptions can improve spatial learning and memory, enhance cognitive exploration, and alleviate anxiety-like behavior in APP/PS1 mice; it also modulate the gut microbiota by restoring its composition and function, reinstating homeostasis, and regulating metabolic pathways—such as amino acid, secondary bile acid, carbohydrate, and sugar metabolism—thereby suggesting translatable microbiota-mediated mechanisms against AD pathology. While APP/PS1 mice recapitulate key gut microbiota alterations seen in human AD (e.g., elevated F/B ratio, reduced SCFA producers), future clinical studies should validate whether DSS-induced microbial remodeling translates to improved cognition and reduced neuropathology in AD patients.

## Data Availability

The raw sequencing data presented in this study are deposited in the NCBI SRA under BioProject accession PRJNA1295736.
